# Shortage and inequalities in the distribution of specialists across community health centres in Uttar Pradesh, 2002–2012

**DOI:** 10.1186/s12913-019-4134-x

**Published:** 2019-05-24

**Authors:** Aditya Singh

**Affiliations:** Independent researcher, Sitapur, Uttar Pradesh India

**Keywords:** Human resources for health, Specialists, Community health centres, India, Uttar Pradesh, Health worker shortage

## Abstract

**Background:**

The onus of providing affordable access to specialist services in rural India primarily lies with publicly funded rural hospitals, also known as community health centres (CHCs). However, no studies have attempted to measure the change in the shortage and distributional inequalities of specialists in the publicly funded rural hospitals of Uttar Pradesh (India). This study attempts to fill that gap.

**Methods:**

The study uses data from the three latest rounds of the District-Level Household Survey, covering a period of 10 years spanning from 2002 to 2012. Shortages were measured against the Indian Public Health Standards for CHCs, and inequalities were measured using Gini and Theil indices, with the latter decomposed to reveal the source of the inequalities. Negative binomial regression was applied to examine the association between facility characteristics and the availability of specialists in CHCs.

**Results:**

The current shortage of specialists stands at 80.7% of the total requirement. Currently, 62.1% of CHCs are functioning without a specialist. The distribution of specialists across CHCs has become progressively uneven over the study period, as shown by the rise in the Gini index (from 0.41 in 2002–2004 to 0.74 in 2012–2013). Decomposition analysis reveals that the contribution of within-district inequalities to overall inequality remains high (85.4% of total inequality). About 50% of within-district inequality is contributed by only 20 districts, most of which belong to eastern and central Uttar Pradesh. The analysis of factors affecting the distribution of the current specialist workforce revealed that the number of available specialists at a CHC is positively associated with the availability of residences for doctors and regular electricity supply, and negatively associated with CHC location and the distance of the CHC from the district headquarters.

**Conclusion:**

The findings suggest that Uttar Pradesh not only needs to recruit more specialists, but it also requires proper implementation of deployment and retention policies to ensure equitable access to specialist care for rural populations. Ensuring the availability of quality accommodations and basic amenities at all CHCs, as well as adequate transport and rural allowance, could help increase the chances of specialists staying in rural and far-off CHCs.

## Background

Government health services in rural India are mainly provided through a three-tier hierarchy of publicly funded health facilities, with community health centres (CHCs) – 30-bed, 24 × 7 rural hospitals that serve about 120,000 people – at the top, primary health centres (PHCs) in the middle, and health sub-centres (HSCs) at the bottom [1]. While HSCs and PHCs provide essential healthcare services, the CHC provides specialist services and acts as a referral centre for the PHCs and HSCs in its catchment area. It provides specialist services to deal with surgical, paediatric, obstetric, and gynaecological emergencies through a team of healthcare professionals that includes four specialist doctors (doctors with a postgraduate medical degree in specific areas), namely surgeon, paediatrician, obstetrician, and anaesthetist [2].

Since private health facilities that offer specialists services are mostly found in urban areas, the onus of providing access to specialist services in rural areas primarily lies with CHCs [3]. The shortage and unequal distribution of a specialist workforce at the CHC level can have serious implications not only for maternal and child mortality, but also for the quality of health-service delivery at higher levels, such as in district or regional hospitals [4]. Since the rural poor mostly rely on publicly funded facilities for their healthcare needs, the lack of specialists at the CHCs often forces them to either forgo treatment or avail specialist services from the private sector, which is infamous for its exorbitant fees [5, 6]. The lack of CHC specialists may cause higher disease burden and mortality for the rural population, and it could also trap rural households into a vicious poverty cycle due to the high out-of-pocket expenditures faced when seeking medical care [5].

The lack of specialists in CHCs also forces the rural poor to seek specialist services from higher-level, publicly-funded hospitals (such as district hospitals and medical colleges) or other CHCs in proximity [7]. The influx of those patients who should have been treated at their local CHC, into the higher-level facilities that are meant for complex and serious cases or in other CHCs, often results in overcrowding, long queues, delays in treatment, and untimely deaths [8]. The lack or unequal distribution of the health workforce (for instance, specialists across CHCs) could also lead to the ineffective use of physical infrastructure and equipment, rendering the invested infrastructure and equipment useless [9]. Hence, it is important to ensure that all CHCs are equipped and staffed with a team of four specialists, as recommended by the Indian Public Health Standards (IPHS) of the Government of India.

The state of Uttar Pradesh (UP), home to over 200 million people, is the largest state in India in terms of population size [10]. It is usually ranked lower than the other states in terms of health- and mortality-related indicators, especially in maternal and child mortality. For instance, the current maternal mortality ratio (MMR) (300 maternal deaths per 100,000 births) and infant mortality rate (IMR) (50 deaths per 1000 births) is about same as the MMR and IMR of many underdeveloped and poor African countries [11]. To improve the situation and reduce the level of burden placed on higher-level facilities, the state of UP not only needs a strong pool of doctors, nurses, and midwives for PHCs and HSCs but also requires a strong and effective specialist workforce at the rural CHCs.

Although the state has rapidly expanded its network of rural health facilities in the last decade, and the number of CHCs in the state has more than tripled [12], little is known about the changes that have occurred in the size and distribution of the health workforce at CHC level in UP. The focus of previous studies on the health workforce in India has been primarily on doctors, nurses, and midwives, and specialist doctors have never received due attention [13–17]. Much of the past literature that measured distributional inequalities and shortages in specialists have emerged from developed countries [18–20].

The aim of this study, therefore, is to measure the change in the shortage and inequality in the distribution of a specialist workforce across publicly funded rural hospitals in UP, while accounting for the sources of those inequalities during a period of about 10 years (between 2002 and 2004 and 2012–2013), which is when major health system reforms were implemented and the number of CHCs had increased significantly within the state [21]. This paper also aims to identify the factors associated with variation in the availability of specialists across CHCs during the most recent period for which data are available (i.e., 2012–2013).

## Methods

### Data source

The data for this study are from the three latest rounds (2002–04, 2007–08, and 2012–13) of a series of nationally representative surveys known as the District-Level Household and Facility Survey (DLHS). All Facility Surveys included in this study collected information on the available resources (health personnel, physical infrastructure, medical equipment, and drug availability) at all CHCs in the state of UP. This study uses information on 257 CHCs from DLHS-2, 693 CHCs from DLHS-3, and 920 CHCs from DLHS-4. The number of CHCs in the state has increased over the years due to expansion of health facilities in rural areas. The details of the sampling strategy adopted for the survey, questionnaires, response rate, and so on can be found in national reports that are publicly available at http://rchiips.org/. This study is based on anonymous public use dataset with no identifiable information on the survey participants. The data can be requested from http://www.iipsindia.ac.in/.

### Statistical analysis

The shortage of specialists is estimated against the norms set by the Indian Public Health Standards (IPHS). The IPHS requires each CHC to be equipped with four specialists – namely, surgeon, paediatrician, obstetrician, and anaesthetist. Thus, the minimum and maximum number of specialists in a CHC should ideally vary between zero and four, respectively.

The summary measures of inequality used in this study are the Gini coefficient (with Lorenz curve) and Theil’s ‘T’. The Gini index is a well-known and widely used measure of inequality that measures aggregate-level inequality [13]. The Gini coefficient varies between 0 and 1, with higher values indicating higher levels of inequality. Since its upper and lower bounds are fixed, the magnitude of inequality that it measures can be easily understood and compared (Anand, 2010). The Gini (G) can be expressed as:

$$ G=\frac{2\  covar\ \left(y,{r}_y\right)}{N\overline{y}} $$

Where *covar* (*y*, *r*_*y*_) is the covariance between the numbers of specialists at CHCs (*y*) and rank of the CHCs according to the number of specialists available (*r*_*y*_) ranging from poorest CHC (rank = 1) to the richest (rank= *N*). *N* is the total number of CHCs and $\overline{y}$ =average number of specialists per CHC.

The Gini index is non-decomposable (i.e., the Gini index is not equal to the sum of the Gini coefficients of its subgroups) [22]. Conversely, Theil’s T is additively decomposable, which means that it can account for different sources of inequality [23]. This index has been widely used in the past since it is a perfectly decomposable measure of inequality [13, 15, 24–27]. The values of Theil’s T vary between 0 and ∞, with 0 representing an equal distribution and higher values representing a higher level of inequality [28]. Theil T can be decomposed into ‘between’ and ‘within’ inequalities. Decomposition involves a partition of units (CHCs) into mutually exclusive and exhaustive groups (such as districts in the state), as well as a calculation of two separate components of overall inequality: a weighted sum of inter-CHC inequality within each district, called the “within-group” inequality, and a “between-group” component that measures inequality that is solely due to variations in specialists’ density across districts [13].

If there are *m* districts in a state, the decomposable Theil index can be expressed as –

1 $$ \boldsymbol{Theil}\ \boldsymbol{T}\  or\ \boldsymbol{GE}\ (1)=\sum \limits_{i=0}^m{s}_i{T}_i\ \sum \limits_{i=0}^m{s}_i\ln \left(\frac{{\overline{y}}_i}{\overline{y}}\right) $$

Where, *s*_*i*_ is specialist share of the district *i* (total number of specialists in the district/total number of specialists in the state), ${\overline{y}}_i$ is the average number of specialists per CHC in the district *i,* $\overline{y}$ is the average number of specialists per CHC in the state.

*T*_*i*_ in the Eq. 1 is the Theil index for the district *i* and can be written as –

2 $$ {T}_i=\frac{1}{N}\sum \limits_{i=1}^N\frac{y_i}{{\overline{y}}_i}\ln \left(\frac{y_i}{{\overline{y}}_i}\right) $$

Where, N is the total number of CHCs in the district *i*, *y*_*i*_ is the total number of specialist available at a CHC in the district *i*, ${\overline{y}}_i$ is the average number of specialists per CHC in the district *i.*

The first part of the Eq. 1 represents “within-group” or “within-district” inequality. The second part represents “between-group” or “between-district” inequality. The ‘between’ inequality values for districts could be either negative or positive, depending on their average number of specialists per CHC compared to average number of the specialists per CHC in the state. A negative value for a district indicates a lower number of specialists available per CHC in that district when compared to the number of specialists available at a typical health facility in the state. The ‘within’ inequality values are always positive. The combined value provides the net contribution of each district to overall inequality. Again, the net (within and between) contribution of each district could be negative or positive, and the total values comprise the overall Theil’s T, which is always positive [15]. For a detailed example of the calculations that have been made in this study to arrive at Theil index, please refer to Appendix. The unit of analysis is CHC. Stata command *ineqdeco* has been used for calculating decomposable Theil T and Gini [29].

Negative binomial regression has been used to examine the association between specialist availability at a given CHC and the facility’s characteristics. The dependent variable, ‘the total number of specialists at a CHC’, is a count variable with a mean of 0.74 and a variance of 1.47. Since the dependent variable consists of non-negative count data, the negative binomial regression technique was chosen for the analysis [30]. The possibility of using a Poisson regression model is ruled out given its strict assumption that the mean and variance of the dependent variable should be same [31]. As mentioned above, the mean of the dependent variable in this study is not equal to its variance; it exhibits a clear over-dispersion. In such cases, a Poisson regression model usually produces inefficient estimates. Conversely, a negative binomial model does not require assumptions of mean and variance equality; it also allows for unmeasured characteristics that generate over-dispersion in the count data [32]. Hence, a negative binomial model was preferred over a Poisson model for this study.

The negative binomial regression model used in this study can be written as follows:

3 $$ {Y}_{fd}={\beta}_0+{\beta}_1{X}_1+{\beta}_2{X}_2+{\beta}_3{X}_3+\dots +{\beta}_n{X}_n $$

Where *Y* is the outcome variable (the total number of specialists at the CHC *f* in district *d*) and X_*1,*_ X_*2,*_ X_*3…*_ X_*n*_ represent facility characteristics that could affect the outcome. The results are presented in the form Incidence Rate Ratios (IRRs). The model was tested to determine potential multicollinearity among the independent variables using variance inflation factor (VIF) as a post-estimation procedure. The overall VIF for the final model is very small (1.79), indicating that there exists no significant multicollinearity among the independent variables.

Over dispersion is estimated using a parameter called alpha. If alpha is zero, then the data are not over-dispersed and a Poisson model is suitable. If alpha is greater than zero, then the data are over-dispersed and the negative binomial distribution models the data more accurately than the Poisson distribution. To test that the dispersion parameter alpha is equal to zero, a likelihood ratio chi-square test was applied, the results of which are also presented along with the model. The large test statistics with very small *P*-values suggest that the response variable is over-dispersed and is better estimated using a negative binomial model than a Poisson model.

The analysis was conducted using Stata (version 12.0) statistical software [33] and the command used for regression analysis was *nbreg*.

Health worker availability in a health facility could be influenced by a host of complex and interconnected factors, linked to health workers’ characteristics and preferences and related to health systems organization and wider social, political and economic environment. Most commonly reported factors include: inappropriate pre-service training for rural and remote areas practice, lack of prospects for further training and career development, low salaries, poor working environments, limited availability of equipment and drugs, insufficient family support, inadequate management and unsupportive supervision. Although the choice of variables included in this study is guided by the existing literature [34–41], the study could include only a limited number of variables that are available in the DLHS-4 dataset. A detailed description of the variables included in the analysis is given in the table below (Table 1).

**Table 1:** Operational definition and categorization of variables

Variables	Description
Dependent variable	
Availability of specialists	The total number of specialists available at a CHC –varies from 0 to 7
Independent variables	
Number of workers	Total number of health worker available at CHC (except specialists) - < =10; 11–20; 21–30; 30+
Residence	Total number of residences available for specialists - None; One; Two; Three; Four
Water	The main source of water at CHC - Tap (piped water supply); Others (bore well, tube well or traditional well); None
Electricity	Whether the CHC has a three-phase electricity connection - Yes; No
FRU	Whether the CHC is designated as First Referral Unit - Yes, No
Location	Where is the CHC located? Rural area; Urban area.
Distance to DH	The distance of CHC from the district headquarters (DH) - < =5 kms; 6–10 kms; 11–30 kms, 31–50 kms; 50+ kms.

## Results

### Shortage of specialists at CHCs

Table 2 provides the details of the overall shortage of specialists at the CHCs over a decade spanning 2002–2004 and 2012–2013. The total number of specialists required for CHCs in UP during 2002–2004 was 1024 – almost two and half times higher than the available number of specialists (*n* = 471) at that time. Although the availability of specialists at the CHCs increased by about 50% during the study period, the overall shortage of specialists, which was about 54% of the total requirement in 2002–2004, had shot up to 81% in 2012–2013; overall, the shortage of all the different specialists grew to reach above 80%. The biggest shortage was recorded for anaesthetists, which held true for all three time points included in this study. However, the shortage of surgeons witnessed the greatest surge, as it grew from 36% in 2002–2004 to 84% in 2012–2013. A similar surge was recorded in the case of paediatricians as well; however, the shortage of obstetricians and anaesthetists, which was already considerably high in 2002–2004, rose by only about 10–12 percentage points over these 10 years.

**Table 2:** Requirement, availability and shortage of specialists at community health centres in Uttar Pradesh, India, 2002–13

Period		Surgeon	Paediatrician	Obstetrician	Anaesthetist	Total
2002–04	Available	163	144	101	63	471
	Required	256	256	256	256	1024
	Shortage	93	112	155	193	553
	Shortage (%)	36.3	43.8	60.5	75.4	54.0
2007–08	Available	202	147	228	127	704
	Required	693	693	693	693	2772
	Shortage	491	546	465	566	2068
	Shortage (%)	70.9	78.8	67.1	81.7	74.6
2012–13	Available	146	180	251	132	709
	Required	920	920	920	920	3680
	Shortage	774	740	669	788	2971
	Shortage (%)	84.1	80.4	72.7	85.7	80.7
IPHS (2012–13)*	Required	1292	1292	1292	1292	5168
	Shortage	1146	1112	1041	1160	4459
	Shortage (%)	88.7	86.1	80.6	89.8	86.3

Note: *IPHS* Indian Public Health Standards (2012); * Population based IPHS norms (2012) require one CHC per 120,000 people; Shortage = Required – Available; Shortage (%) = (Shortage/Required) *100

The population-based IPHS norm requires at least one CHC to be established for every 120,000 people. To fulfil this norm, the state should have 1292 CHCs to serve its 160 million people who live in rural areas. After considering the IPHS norm, the current shortage increases by about another 5% to reach 86%. Similarly, the shortage of surgeons and anaesthetists approached 90% of their total requirement.

### Distribution of specialists

The average number of specialists in the CHCs of UP during 2002–2004 was 1.84. This decreased considerably over the following 10 years to 0.79 specialists per CHC. Table 3 shows the distribution of specialists across CHCs in UP. About 20% of all CHCs did not have even a single specialist, while another 23% of CHCs had only one specialist in 2002–2004. The situation worsened since then, as in 2012–2013, the proportion of CHCs without any specialists increased to 62% in 2012–2013, while the proportion of CHCs with one specialist decreased slightly by four percentage points. The proportion of CHCs with two, three, four, and five or more specialists also reduced substantially during this time.

**Table 3:** Distribution of community health centres by number of specialists in Uttar Pradesh (India) during 2002–13

Year	Type of specialist	Number of specialists					
		0	1	2	3	4	5+
2002–04 (*n* = 256)	Surgeon	37.9	59.8	2.0	0.3	0.0	0.0
	Obstetrician	63.7	34.4	1.5	0.4	0.0	0.0
	Paediatrician	48.4	47.3	3.9	0.0	0.4	0.0
	Anaesthetist	75.4	24.6	0.0	0.0	0.0	0.0
	All specialists	20.3	23.1	23.8	21.9	7.4	3.5
2007–08 (*n* = 693)	Surgeon	72.7	26.0	1.0	0.1	0.0	0.1
	Obstetrician	70.1	27.1	2.5	0.3	0.0	0.0
	Paediatrician	78.2	20.9	0.9	0.0	0.0	0.0
	Anaesthetist	82.7	15.9	1.0	0.4	0.0	0.0
	All specialists	48.3	20.4	16.6	10.0	4.2	0.6
2012–13 (n = 920)	Surgeon	85.5	13.6	0.4	0.3	0.1	0.0
	Obstetrician	80.0	16.7	2.5	0.8	0.0	0.0
	Paediatrician	81.4	17.7	0.8	0.1	0.0	0.0
	Anaesthetist	88.5	10.1	0.8	0.3	0.1	0.2
	All specialists	62.1	19.1	8.2	7.0	2.3	1.4

Note: All figures are in percent. Interpretation – 37.9 should be interpreted as follows – 39.7% CHCs are functioning without any surgeon. 59.8 should interpreted as follows – 59.8% CHCs have one surgeon. ‘n’ is the number CHCs

Among the different categories of specialists, the proportion of CHCs without a specialist have increased substantially during the study period. For instance, the proportion of CHCs without a paediatrician rose from 48% in 2002–2004 to 81% in 2012–2013. The case was similar for the other three specialist types, although the increase in the proportion of such CHCs was not as high as in the case of paediatricians. In 2012–2013, about 14% of CHCs had one or more surgeon, while 20% had one or more obstetrician in position. Paediatricians and anaesthetists were available in 19 and 17% of CHCs, respectively.

Table 4 presents an overview of the placement of available specialist doctors across CHCs during the study period. It was found that of all CHCs that had an available specialist in 2012–2013, 55% of these health centres had only one specialist. The proportion of CHCs that had all four specialists was only about 6%. The proportion of CHCs that had two to three specialists (but no anaesthetist) was about 22%. Thus, one can see that there was – and still is –considerable variation in the distribution of specialists across CHCs. The next section will, therefore, provide details on the distributional inequality of specialists, as measured by widely used measures of inequality – Theil’s ‘T’ and the Gini coefficient.

**Table 4:** Placement of specialist doctors across community health centres in Uttar Pradesh during 2002–04, 2007–08, and 2012–13

Availability of health workers	2002–04 (*n* = 204)	2007–08 (*n* = 358)	2012–13 (*n* = − 349)
Only Anaesthetist	3.4	8.1	7.4
Only Paediatrician	9.8	8.7	18.1
Only Surgeon	12.7	14.0	11.5
Only Obstetrician	3.4	12.0	18.3
Two or more specialists with anaesthetist/s	18.6	19.6	17.2
Two or more specialists with no anaesthetist	43.1	31.8	21.8
All four specialists available	8.8	5.9	5.7

### Overall inequality

Table 5 provides a glimpse of the overall inter-CHC inequality in the distribution of specialists and other categories of health workers (doctors, nurses, and allied health professionals) at CHCs for the study period (i.e., 2002–2013). Overall health worker inequality decreased by about 31% (11 Gini points) during the period. This decrease is visible in Theil’s T as well, which dropped from 0.025 in 2002–2004 to 0.12 in 2012–2013. The same is true for all other health worker categories as well, except for specialists. The inequality in the distribution of specialists rose by about 78% (33 Gini points). Regarding Theil’s T, the unequal distribution of specialists had gone up from a modest 0.34, reaching a high of 1.14. It is interesting to note that the 2002–2004 inequality for doctors and nurses is much lower than that for specialists, but the reverse is true for the latest time point (i.e., 2012–2013). The inequality for specialists in 2012–2013 is considerably higher than the inequality for nurses and doctors.

**Table 5:** Inter-CHC inequality for health professionals in Uttar Pradesh (India) between 2002–04 and 2012–13

Health worker category	Inequality measure	Inequality			Change between 2002–04 and 2012–13	
		2002–04 (*n* = 257)	2007–08 (n = 693)	2012–13 (n = 920)	Absolute	in %
All Health Workers	Theil T	0.25	0.20	0.12		
	Gini	0.38	0.34	0.27	−0.117	−30.5
Doctors	Theil T	0.66	0.27	0.23		
	Gini	0.57	0.35	0.36	−0.210	−37.0
Nurses	Theil T	0.61	0.42	0.29		
	Gini	0.58	0.47	0.39	−0.189	−32.8
Specialist	Theil T	0.34	0.79	1.14		
	Gini	0.41	0.63	0.74	0.325	78.3
Surgeon	Theil T	0.51	1.34	1.98		
	Gini	0.42	0.74	0.87	0.452	108.6
Paediatrician	Theil T	0.70	1.54	1.70		
	Gini	0.52	0.79	0.82	0.298	56.9
Obstetrician	Theil T	1.02	1.24	1.67		
	Gini	0.65	0.73	0.83	0.179	27.5
Anaesthetist	Theil T	1.40	1.80	2.30		
	Gini	0.75	0.84	0.91	0.152	20.1

**Fig. 1:**
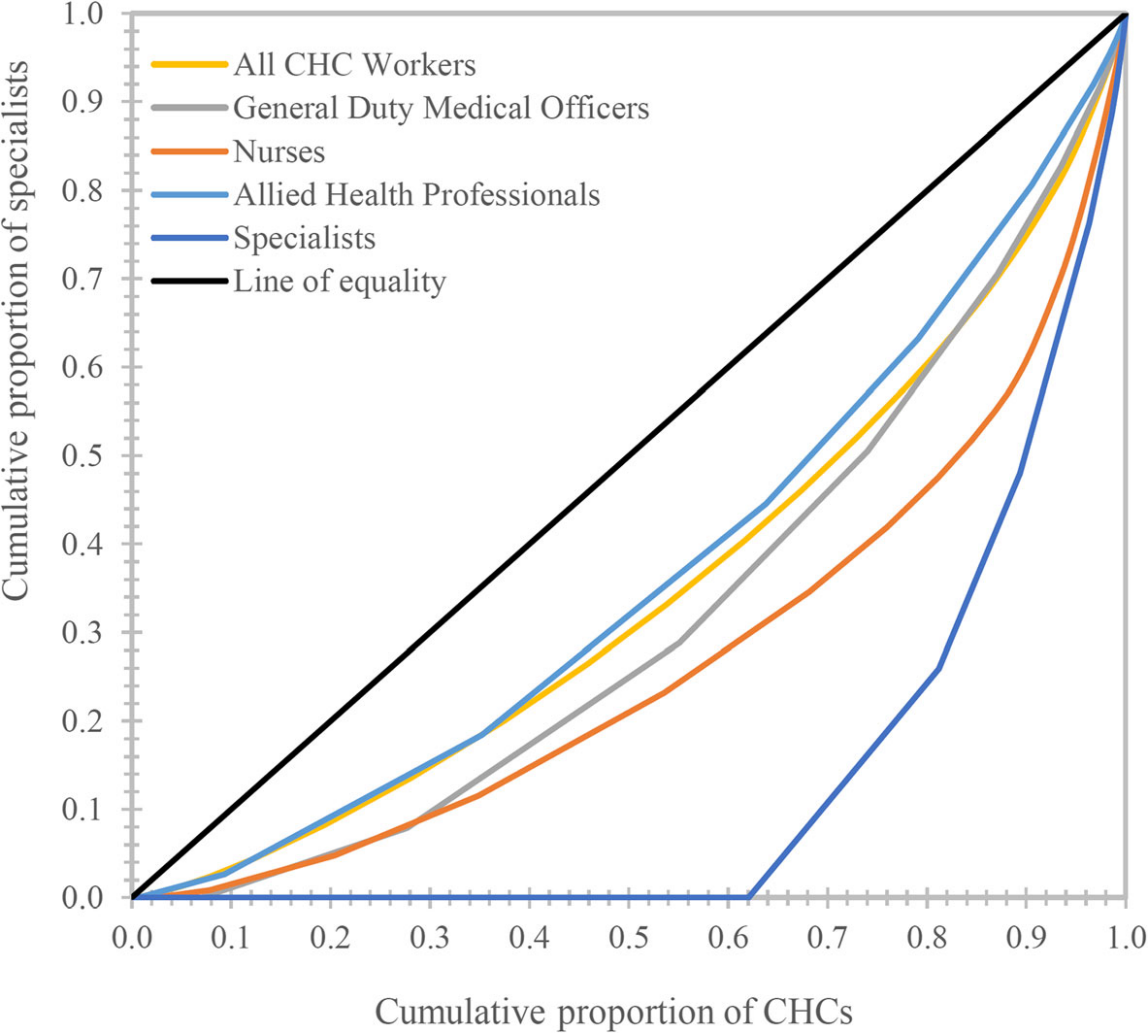
Lorenz curve showing inequality in the distribution of health workers at CHCs in Uttar Pradesh, India, 2012–13 (*n* = 920)

**Fig. 2:**
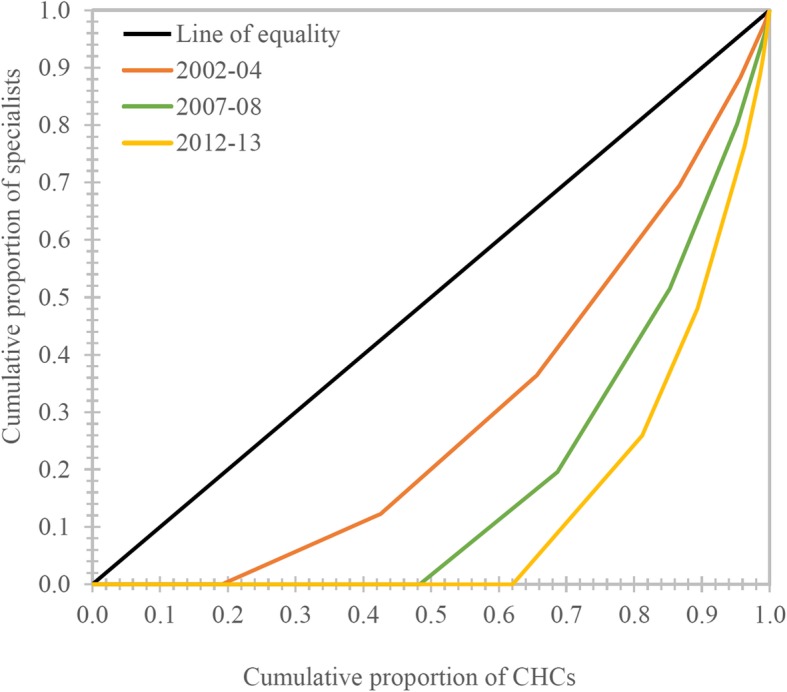
Lorenz curve showing inequality in the distribution of specialists at CHCs in Uttar Pradesh, India, 2002–13

The Gini coefficient for all categories of specialists is above 0.80, which suggests that their distribution is highly unequal. Anaesthetists are the most unequally distributed among all (Theil’s ‘T’ = 2.30; Gini = 0.91). The same is true for their 2002–2004 distribution as well. The biggest change in distributional inequality occurred for surgeons and paediatricians; these groups witnessed an increase of about 109 and 57% in their Gini coefficients, respectively, while the corresponding increase for anaesthetists and obstetricians was only about 20 and 27%, respectively. In this regard, it must be noted that the Gini index for surgeons and paediatricians during 2002–2004 was smaller than that for obstetricians and anaesthetists (Figs. 1 and 2).

Thus, it is evident that the inequality in specialist distribution has risen over this period, while the same is not true for other categories of CHC health workers. The next section presents the results of the decomposition of overall inequality (Theil’s ‘T’) on the current (2012–2013) distribution of specialists into within-district inequality and between-district inequality.

### Decomposition of overall inequality (Theil’s ‘T’)

Table 6 provides the results of the decomposition of Theil’s ‘T’ for specialists into within- and between-district inequalities for all three time points. The between- and within-district values for 2012–2013 are 0.17 and 0.98, respectively, thus indicating that between- and within-district inequalities contribute about 18 and 82% to overall inequality, respectively. Although overall inequality has gone up considerably, the contribution of between- and within-district inequalities to overall inequality has changed only slightly over time. The between-district inequalities witnessed a decline of about 8%.

**Table 6:** Decomposition of inter-CHC inequality by districts

Period	Inequality measure	Overall inequality	Within district inequality	Between district inequality	Within district inequality (% of overall)	Between district inequality (% of overall)
2002–04 (*n* = 257)	Theil T	0.34	0.26	0.09	74.0	26.0
	Gini	0.41				
2007–08 (*n* = 693)	Theil T	0.79	0.65	0.14	81.8	18.2
	Gini	0.63				
2012–13 (*n* = 920)	Theil T	1.14	0.98	0.17	85.4	14.6
	Gini	0.74				

**Fig. 3:**
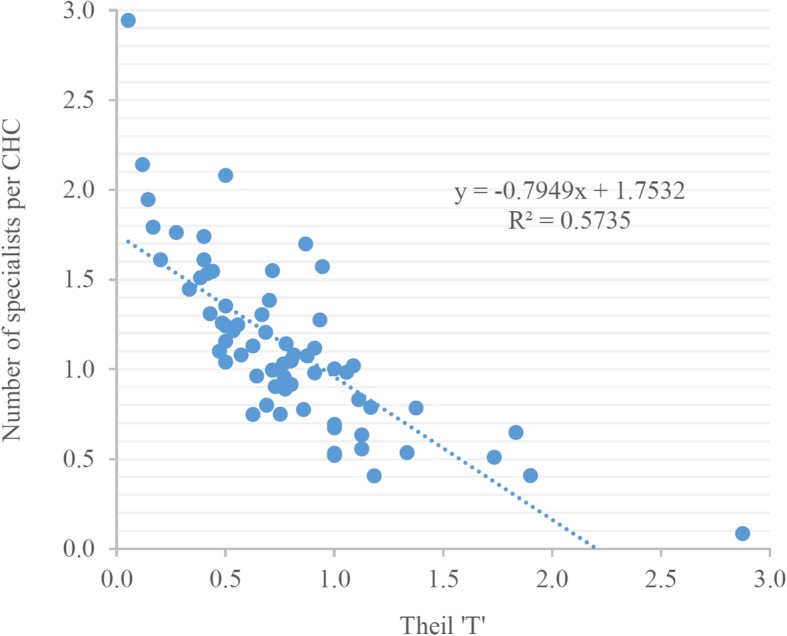
The relationship between the average availability of specialists and the level of inequality in their distribution across 70 districts of Uttar Pradesh, 2012–13

**Fig. 4:**
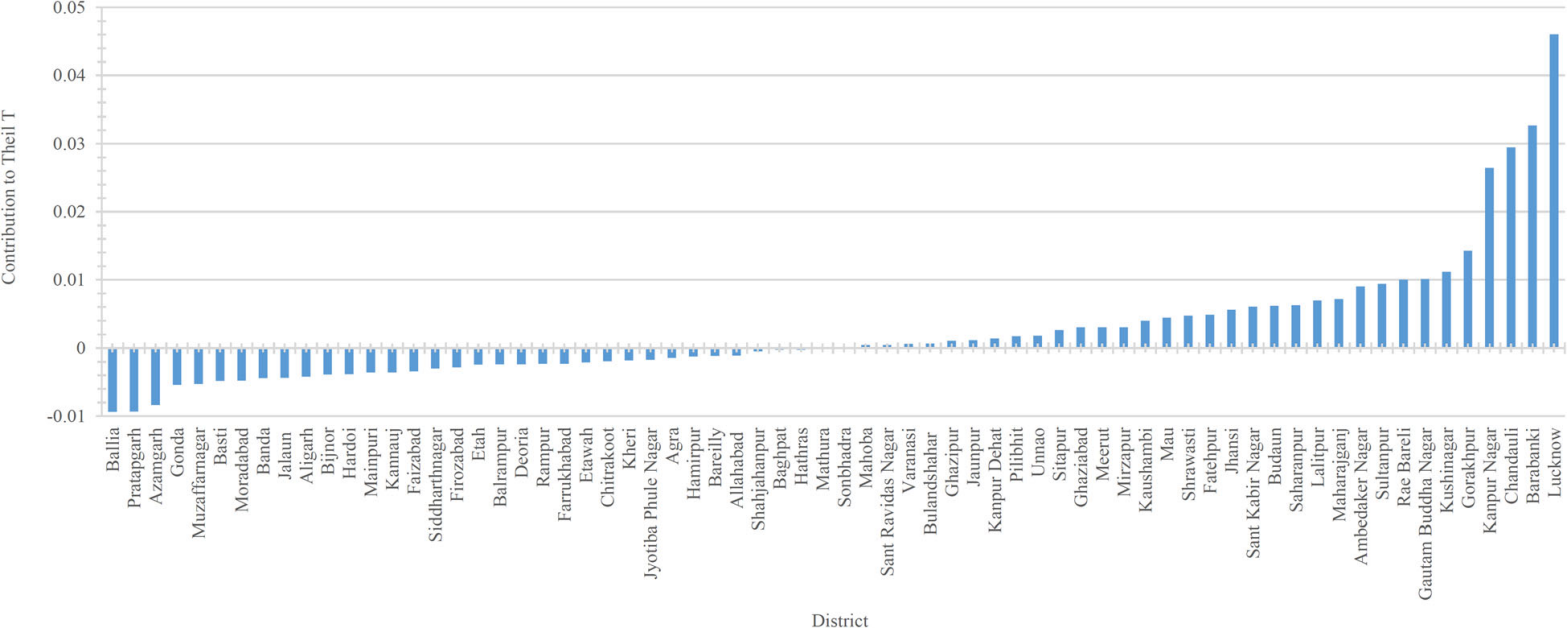
Contribution of districts to the overall ‘between-district’ inequality, Uttar Pradesh, 2012–13 (*n* = 920)

**Fig. 5:**
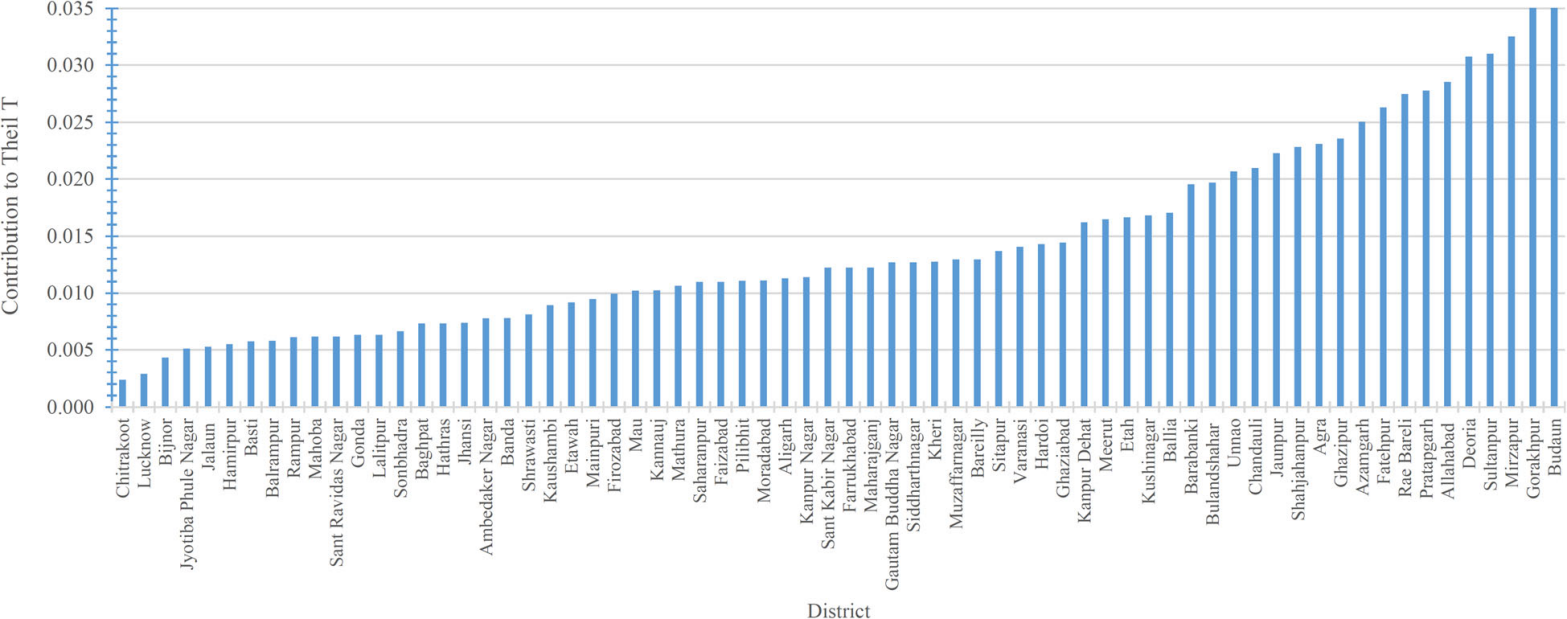
Contribution of districts to the overall ‘within-district’ inequality, Uttar Pradesh, 2012–13 (*n* = 920)

Figures 4 and 5 show the contribution of different districts to overall between- and within-district inequality. The contribution to between-district inequality is expressed in positive and negative values. Graph 4 reveals that the highest contribution to the overall between-district inequality comes from the districts of Lucknow, Kanpur Nagar, Chanduali, and Barabanki, while the highest negative contribution comes from the districts of Ballia and Pratapgarh. As for the contribution of different districts to the overall within-district inequality (Fig. 5), the highest contribution comes from the Badaun and Gorakhpur districts. Of the 70 districts in UP, the top 20 districts contribute about 52% of the total within-district inequality, and all except three belong to the Poorvanchal (Eastern UP) and Avadh region (Central UP). The lowest contribution to overall within-district inequality comes from the Chitrakoot and Lucknow districts. The districts falling on the left-hand side of the graph slightly contribution to the overall within-district inequality. Except for a few districts, most belong to Western UP. The state capital, Lucknow, is a curious case; its contribution to the overall between-district inequality is the highest among all districts in the state (an indication of higher per-CHC availability of specialists), while the contribution to within-district inequalities is the second lowest in the state. Figure 3 shows that there is a strong relationship between the average availability of specialists per CHC and the within-district inequality, as measured by Theil’s ‘T’; Lucknow is an example of such a pattern (Figs. 3, 4 and 5).

### Factors associated with the distribution of specialists in CHCs

#### Distribution of the characteristics of CHCs

Table 7 provides a glimpse into the facility characteristics, such as the number of workers available and the availability of residences, water, electricity, and some other variables (such as the location of a CHC, its first referral unit (FRU) status, and the distance to district headquarters), that could influence the availability of specialists at the CHCs. A little over 50% of CHCs in UP have fewer than ten health workers. A very small proportion of CHCs has ≥30 health workers. About 21% of CHCs receive their water through a piped service, whereas about two-thirds of CHCs use bore wells, tube wells, or traditional wells for their water. About 2% do not have a water source within their premises at all. As for electricity, about 15% are running without a three-phase electricity connection. About one-fifth of all CHCs are designated as a FRU, and about 15% of CHCs are located in areas that are designated as ‘urban’ per the definition provided in the Census of India.

**Table 7:** Results of negative binomial regression, 2012–13 (*n* = 920)

Independent variables	Categories	Number of CHCs	%	Unadjusted		Adjusted	
				IRR	95% CI	IRR	95% CI
Number of workers	30 + ^@^	471	5.3				
	< 10	347	45.8	0.351***	0.225, 0.548	0.570***	0.403, 0.806
	11--20	53	41.2	0.752	0.484, 1.168	0.872	0.625, 1.218
	20–30	49	7.6	0.506**	0.274, 0.934	0.629	0.385, 1.029
Number of residences	None^@^	493	53.6				
	One	125	13.6	4.634***	3.477, 6.177	3.864***	2.934, 5.088
	Two	89	9.7	6.158***	4.531, 8.37	4.755***	3.543, 6.383
	Three	78	8.5	8.574***	6.262, 11.738	6.206***	4.592, 8.387
	Four	135	14.7	4.404***	3.293, 5.891	3.585***	2.703, 4.754
Source of water	Tap^@^	196	21.3				
	Others	707	76.9	0.787	0.603, 1.026	1.105	0.895, 1.365
	None	17	1.9	0.272**	0.084, 0.879	0.639	0.221, 1.846
Electricity connection	Yes^@^	780	84.8				
	No	140	15.2	0.443***	0.307, 0.639	0.719*	0.516, 1.001
FRU	Yes^@^	170	18.5				
	No	750	81.5	0.304***	0.242, 0.382	0.518***	0.428, 0.627
Location	Urban^@^	140	15.2				
	Rural	780	84.8	0.514***	0.389, 0.68	0.695***	0.560, 0.863
Distance to DH	<=5 kms^@^	26	2.8				
	6--10 kms	37	4.0	0.366**	0.146, 0.917	0.473*	0.224, 1.002
	11--30 kms	451	49.0	0.748	0.397, 1.409	0.571**	0.352, 0.927
	31--50 kms	284	30.9	0.791	0.414, 1.511	0.574**	0.350, 0.941
	51+ kms	122	13.3	0.739	0.371, 1.471	0.705	0.413, 1.201

Alpha = .4887607

LR test of alpha = 0: chibar2(01) = 48.83

Prob > = chibar2 = 0.000

Note: *IRR* Incidence rate ratio, *FRU* First Referral Unit, *DH* District Headquarter, *CI* Confidence Interval. @ = Reference Category, Level of significance: * indicates *p* < 0.10, ** indicates *p* < 0.05, *** indicates *p* < 0.01

#### Negative binomial regression results

Table 7 presents the results of the negative binomial regression that has been performed to examine the association of facility characteristics with the number of specialists available in CHCs. Some factors have emerged as statistically significant during the analysis. The results indicate that the total number of health workers at a CHC is a significant predictor of specialist availability. The number of specialists at CHCs with fewer than ten workers is expected to decrease by about 43% with respect to the number of specialists at CHCs with more than 30 workers. Similarly, the number of residences available for specialists at a given CHC is also strongly associated with the number of specialists available at that location. The availability of specialists at the CHCs with one, two, and three residences is expected to be four, five, and six times higher, respectively than the CHCs without any residences. CHCs without a three-phase electricity connection are expected to have fewer specialists than those with a three-phase connection (IRR = 0.719). The rural location and non-FRU status of a CHC are also expected to reduce the number of specialists at a location. Non-FRUs are expected to have about 50% fewer specialists than FRUs (IRR = 0.518). The distance from the CHC to district headquarters is also an important predictor of the availability of specialists at CHCs. Specifically, the further the CHCs is located from the district headquarters, the fewer specialists it is expected to have.

## Discussion

The results indicate that the absolute number of specialists at CHCs in UP has increased from 2002 to 2013; however, the shortage of specialists in relation to the requirement (as based on the number of CHCs) has grown as well. This is due to the fact that the increase in the number of newly established CHCs has been far greater than the increase in the number of specialists recruited during the study period. The current shortage of specialists is about 80% of the total requirement (based on the current number of CHCs in the state). The overall shortage becomes significantly more acute if the population-based IPHS norms are applied to the current situation. The overall scenario indicates that the efforts being made to fill vacant positions for specialists at CHCs in the state are grossly insufficient.

There could be several reasons why the state government has not been able to increase the number of specialists in the system. First, there are simply not enough specialists graduating every year to fill the gap. For instance, the medical colleges in the state (including private ones) have only 93 and 74 postgraduate seats in total in obstetrics/gynaecology and paediatrics courses, respectively [42]. Even if one assumes that all obstetricians will join CHCs upon completion of their courses which, given the current policies, is obviously not going happen, it would still take six to 7 years to fill all vacant positions for obstetricians at the CHCs in UP. The same holds true for other categories of specialists as well.

One study found that the state has been ‘lethargic’ as far as recruitment of health workers is concerned [43]. It also argues that one of the reasons why the state is not able to recruit enough people could be the lack of candidates applying for the Provincial Medical Services, the method through which specialists in the state are recruited for their service in public health facilities [44]. It has been noted that a lot of those who do apply and get selected for these positions do not show up for the interview due to the lengthy recruitment process, leaving many posts vacant [45]. Since the state does not have a provision of waiting lists for regular appointments, there is no way to fill the posts left vacant by those who prefer not to join the services [43]. Many previous studies have found that specialist doctors are also recruited against the post of ‘medical officer’. Such policy loopholes further aggravate the problem of specialist shortages in the system [44].

In the past, the state has tried to fill specialists positions through short-term contractual appointments, but this has been done with limited success [45]; in fact, only a few seats have been filled in the last 10 years [46]. A recent study has noted that district authorities who were in charge of implementing this measure were not very keen to take up such measures [4]. Although the state government has invited retired specialists to re-join the system this year to provide their services, the specialists’ deployment is limited only to District Hospitals [47]; CHCs do not seem to be the priority at the moment.

Previous studies in other Indian states have found that most specialists, after they finish their studies, prefer private practice rather than joining public health system; this is done primarily for economic reasons [48]. Currently, the state of UP does not have strong financial and non-financial incentives for working in rural areas. Even those that do exist are too weak to offset the opportunity costs of working in the private sector [48–50]. Another obstacle associated with recruiting specialists is that the policy allows only those doctors who are registered in UP to join the system. Thus, specialists registered in other states cannot become a part of the state’s public health system [4, 43]. In addition, the huge shortage of a specialist workforce in the state is also the result of a lack of vision for the health workforce in rural public health facilities. It is surprising that the state still does not have a framework for medium- or long-term human resource for health (HRH) planning for its rural health system [51]. Moreover, the state also lacks a central HRH database that could help policymakers to keep an eye on HRH dynamics in the public health system [52].

Similar to previous small-scale studies, this study also found that a huge number of CHCs do not have the correct combination of specialists to provide emergency obstetric care or to conduct surgical interventions, such as caesarean section [51]. While some CHCs have a gynaecologist, but not an anaesthetist, there are other CHCs where the latter is posted without any surgeon. Also, there are many CHCs where an anaesthetist is posted alone without any paediatrician surgeon or obstetrician [4]. Such misallocation of the specialist workforce across CHCs can cause gross underutilization of the skills of specialist doctors, while also constituting a waste of financial resources [53]. Therefore, the government of UP should focus not only recruiting more specialists, but also on redistributing the existing specialist workforce in correct combinations to fully utilise their services.

The inequality analysis revealed that while the inequalities for doctors and nurses have decreased, the inequality in the distribution of specialists has increased during the study period (i.e., 2002–2013). The decomposition analysis indicated that the share of within-district inequality has always been higher than between-district inequality. It is interesting to note that the share of within-district inequality has risen steadily over the period. The current share of within-district inequality in terms of total inequality is about 85%; therefore, the strategies to reduce inter-CHC inequality in the distribution of specialists should focus on those districts that contribute the most to overall within-district inequality. Several districts in the Poorvanchal (eastern UP) and Avadh (central UP) regions contribute heavily to the overall within-district inequalities. Most of these districts are characterised by low maternal and child healthcare utilisation when compared to the rest of the districts in the state [54]. They also lag behind in terms of socio-economic development [55]. Thus, the situation of these districts echoes what is known as Hart’s ‘law of inverse care’, which states that the availability of good medical care tends to vary inversely with the need for it in the population served [56]. Therefore, the need of the hour is to update recruitment and deployment policies, so that they not only focus on improving the overall availability of specialists through increased recruitment activities in these districts, but also on equitable deployment of specialists within these districts.

The current distribution of specialists across CHCs could be the result of a number of factors ranging from recruitment and deployment policies of the state, to the personal choices made by doctors who, themselves, are affected by several factors such as working environment, amenities, availability of a residence, and travel times, and so on [57, 58]. A recently conducted study revealed that most postings for doctors in the state are highly politicized and it is not unusual for doctors or health workers to use their political influence to cherry-pick their desired facility. The unavailability of doctors in many facilities across the state makes it even easier for them to get transferred to a place of their choice [44]. As a result, the facilities with the greatest need – largely those that are in far-off and poorly connected rural areas – are often neglected.

Utilizing the variables available in the latest dataset, this study also examined whether these variables are associated with specialist availability at the CHCs in the state. The results of the analysis revealed that the CHCs with a low number of health workers (fewer than 10) are expected to have a low number of specialists. It is possible that those CHCs with a low number of health workers face a higher burden of work that forces specialists to move out to health facilities staffed with higher numbers of health workers, thus experiencing relatively lower burden [59, 60]. The results of the regression analysis also revealed that those CHCs that had residences available for specialists are expected to have a higher number of specialists than those CHCs that do not have residences for specialists. These results are in line with those of several previous studies conducted in developing countries [48, 61, 62]. For instance, several respondents from a study conducted in Malawi reported that poor housing conditions at the hospital served a factor that lead to demotivation; as such, these specialists left their jobs [63]. The same was found to be true for doctors from Ghana, who reported that they were willing to take rural posts, but the lack of work-based accommodations and delayed renovations forced them to give up a prospective rural post, and so they settled in a city instead [36]. It should be noted that over half of the CHCs in the state do not have residences for specialists.

Many previous studies showed how the availability of basic amenities, such as water and electricity, are regarded as necessary by health workers; similarly, the results of this study also showed that the lack of electricity connection is associated with a decrease in specialist availability. The supply of electricity in the state is highly regular and the rural areas receive electricity for only 6 h a day, on average [64]. The urban life (electrical appliances are an integral part of daily life in urban areas) which specialist doctors are accustomed of and wish to lead, is nearly impossible in such rural settings. Given that a considerable number of CHCs, especially the recently commissioned ones, in the state are often located in the outskirts of the main settlement, not having a regular supply of electricity poses a serious security threat, as a lack of lighting, especially during the night, makes them vulnerable to violence, theft, and burglary [50].

In this study, the location of a CHC has been found to be a significant predictor of the number of specialists available at a CHC. It turns out that the CHCs located in rural areas of the state are likely to have fewer specialists than their urban counterparts, regardless of their FRU status. This finding is in tune with those of many previous studies, which have found that rural and remote health facilities are often poorly equipped and inadequately supplied with drugs [65], their physical working conditions are severe [49], the career growth opportunities are fewer [50], and staff are poorly supported or supervised and often feel isolated and neglected [66].

The unavailability of specialists in rural areas could be attributed to a number of push factors such as poor road connectivity, an unreliable public transport system, a lack of quality schooling for children, and so forth, that repel health workers from rural and remote areas [41, 57–59, 67, 68]. Most rural areas in the state have poor road connectivity and public transport is almost non-existent [69]. In such situations, the use of private vehicles becomes necessary. Since the state government does not provide any transportation or rural allowance [70] to travel to those CHCs located in rural and far-off areas, it is not difficult to understand why rural CHCs have fewer specialists when compared to their urban counterparts, which are well connected to district headquarters or major cities through major district roads and state highways. The unavailability of quality schooling for children in rural areas is another issue that is often quoted as a reason for not joining/leaving rural health services [67, 71]. The desire for specialist doctors to live with their families has also been noted as a reason for the lack of specialists in the rural public health system. In this regard, a recent study conducted in four North Indian states has found that a considerable proportion of specialists are willing to leave rural CHCs to live with their families in towns, cities, or district headquarters [4].

The study has several strengths. First, it contributes to the scant literature available on the issue of HRH shortage and the inequalities in the publicly funded health system in rural India. It is probably the first study to delve into the issues associated with specialist distribution in the public health system of UP. Unlike previous studies that featured small sample sizes, the present study included all rural hospitals of the state in question. Therefore, the reliability of the results is not an issue, and the findings can be used to inform policy development. Furthermore, the decomposable inequality measure applied in this study is a widely used and tested method in the field of HRH inequalities. The limitations of this study must be noted as well. First, given the cross-sectional nature of the dataset, casualty cannot be inferred. The relationship in the regression analysis may suffer from reverse casualty. It is possible that those CHCs where specialists are posted receive more resources and better facilities, or vice versa. Second, several factors such as personal preferences, working conditions and organizational environment, incentives, career growth opportunities, etc. that may affect the distribution of specialists across CHCs were not included in the analysis given the lack of such variables in the dataset. Third, the analysis does not address the situation after 2013. This is because the unit-level data on specialists for 2013 onwards is not available.

The present study has highlighted some critical issues related to the shortages and distributional inequalities of the specialist workforce in the Indian state of UP. Future research could undertake similar analysis for other Indian states; they can also analyse whether areas with higher numbers of specialists in the public health system are more likely to have better maternal and child health outcomes. Such an analysis could help policymakers decide upon the future roadmap to achieve maternal- and child-health-related sustainable development goals [26, 72].

## Conclusion

The CHCs in the state are not only suffering from a huge shortage of specialists, but also from deep distributional inequalities in the existing workforce. The situation has become progressively worse over the study period. The shortage of specialists has shot up from 80 to 85% of the total requirement, and the inequality in their distribution has grown considerably as well. Decomposition analysis revealed that a large part of the overall inequality in the distribution of specialist doctors stems from within-district inequality. Moreover, the contribution of within-district inequality to overall inequality has steadily grown over time. The HRH policies in the state should thus focus not only on recruiting more specialists, but also on finding innovative strategies – such as rotation postings or higher monetary incentives for difficult and far-off areas – to influence the within-district distribution of specialists to make it more equitable. The focus should be on those districts from eastern UP where resources are highly concentrated in only a few CHCs. It has also been noted that rural CHCs, on average, have fewer specialists when compared to their urban counterparts. Therefore, targeted attempts should be made to deploy and retain a specialist workforce in rural CHCs. Apart from recruiting and deploying more specialists, the government should ensure the availability of basic facilities such as water, electricity, and quality accommodations for doctors at the CHCs, especially for those CHCs that are in interior locations and where commuting to the city is not possible. Future health workforce policies and interventions in the state should thus focus on increasing specialist availability in rural CHCs. From the discussion, it is clear that the state lacks strong monetary and non-monetary incentives/rewards for rural service that could stave off the negative effects of the rural location of the CHCs. This is a policy gap that the state government needs to consider immediately.

## Appendix

**Table 8:** Computing Decomposable Theil ‘T’: An Example

CHC S.N.	District	# of specialists (*y*_*i*_)	Share of specialists (*s*_*i*_)	Average # of specialists in district (${\overline{y}}_i\Big)$	$\frac{{\overline{y}}_i}{\overline{y}}$	$\ln \left(\frac{{\overline{y}}_i}{\overline{y}}\right)$	Between-district inequality values for districts ${s}_i\ln \left(\frac{{\overline{y}}_i}{\overline{y}}\right)$	$\frac{y_i}{{\overline{y}}_i}$	$\ln \left(\frac{y_i}{{\overline{y}}_i}\right)$	$\frac{y_i}{{\overline{y}}_i}\ln \left(\frac{y_i}{{\overline{y}}_i}\right)$	District Theil T *T*_*i*_	Within-district inequality value for districts *s*_*i*_*T*_*i*_
1	South	3						0.68	−0.38	−0.26		
2	South	4						0.91	−0.10	−0.09		
3	South	5						1.14	0.13	0.15		
4	South	6						1.36	0.31	0.42		
5	South	4						0.91	−0.10	−0.09		
	Total South	22	0.36 (22/61)	4.4 (22/5)	1.15	0.14	0.05			$\sum \limits_{i=1}^N\frac{y_i}{{\overline{y}}_i}\ln \left(\frac{y_i}{{\overline{y}}_i}\right)=$0.13	0.03	0.01
6	North	3						1.25	0.22	0.28		
7	North	1						0.42	−0.88	−0.36		
8	North	2						0.83	−0.18	− 0.15		
9	North	1						0.42	−0.88	−0.36		
10	North	5						2.08	0.73	1.53		
	Total North	12	0.20 (12/61)	2.4 (12/5)	0.63	−0.46	−0.09			$\sum \limits_{i=1}^N\frac{y_i}{{\overline{y}}_i}\ln \left(\frac{y_i}{{\overline{y}}_i}\right)=$0.93	0.19	0.04
11	West	3						0.67	−0.41	−0.27		
12	West	5						1.11	0.11	0.12		
13	West	2						0.44	−0.81	−0.36		
14	West	7						1.56	0.44	0.69		
15	West	9						2.00	0.69	1.39		
16	West	1						0.22	−1.50	−0.33		
	Total West	27	0.44(27/61)	4.5 (27/6)	1.18	0.17	0.07			1.23	0.20	0.09
	Total All CHCs	61										
Average number of specialist per CHC in the province		3.81 $\Big(\overline{y}$)	Overall between-district inequality $\sum \limits_{i=0}^m{s}_i\ln \left(\frac{{\overline{y}}_i}{\overline{y}}\right)$ 0.03			Overall within-district inequality $\sum \limits_{i=0}^m{s}_i{T}_i$ 0.14		Overall Theil T $\sum \limits_{i=0}^m{s}_i{T}_i+\sum \limits_{i=0}^m{s}_i\ln \left(\frac{{\overline{y}}_i}{\overline{y}}\right)$ 0.17				

## Abbreviations

CHC: Community Health Centre
DLHS: District Level Household Survey
FRU: First Referral Unit
HRH: Human Resource for Health
HSC: Health Sub-Centre
IMR: Infant Mortality Rate
IPHS: Indian Public Health Standards
IRR: Incidence Rate Ratios
MMR: Maternal Mortality Ratio
PHC: Primary Health Centre
UP: Uttar Pradesh
VIF: Variance Inflation Factor
